# Assessing heat effects on respiratory mortality and location characteristics as modifiers of heat effects at a small area scale in Central-Northern Europe

**DOI:** 10.1097/EE9.0000000000000269

**Published:** 2023-09-13

**Authors:** Sofia Zafeiratou, Evangelia Samoli, Antonis Analitis, Antonio Gasparrini, Massimo Stafoggia, Francesca K. de’ Donato, Shilpa Rao, Siqi Zhang, Susanne Breitner, Pierre Masselot, Kristin Aunan, Alexandra Schneider, Klea Katsouyanni

**Affiliations:** a Department of Hygiene, Epidemiology and Medical Statistics, Medical School, University of Athens, Athens, Greece; b Department of Public Health, Environments and Society, London School of Hygiene & Tropical Medicine, London, United Kingdom; c Department of Epidemiology, Lazio Region Health Service (ASL ROMA 1), Rome, Italy; d Division for Climate and Environment, Norwegian Institute of Public Health (NIPH), Oslo, Norway; e Institute of Epidemiology, Helmholtz Zentrum München (HMGU), Neuherberg, Germany; f CICERO Center for International Climate Research, Norway; g Environmental Research Group, MRC Centre for Environment and Health, Imperial College, London, United Kingdom

## Abstract

**Background:**

Heat effects on respiratory mortality are known, mostly from time-series studies of city-wide data. A limited number of studies have been conducted at the national level or covering non-urban areas. Effect modification by area-level factors has not been extensively investigated. Our study assessed the heat effects on respiratory mortality at a small administrative area level in Norway, Germany, and England and Wales, in the warm period (May–September) within 1996–2018. Also, we examined possible effect modification by several area-level characteristics in the framework of the EU-Horizon2020 EXHAUSTION project.

**Methods:**

Daily respiratory mortality counts and modeled air temperature data were collected for Norway, Germany, and England and Wales at a small administrative area level. The temperature-mortality association was assessed by small area-specific Poisson regression allowing for overdispersion, using distributed lag non-linear models. Estimates were pooled at the national level and overall using a random-effect meta-analysis. Age- and sex-specific models were also applied. A multilevel random-effects model was applied to investigate the modification of the heat effects by area-level factors.

**Results:**

A rise in temperature from the 75th to 99th percentile was associated with a 27% (95% confidence interval [CI] = 19%, 34%) increase in respiratory mortality, with higher effects for females. Increased population density and PM_2.5_ concentrations were associated with stronger heat effects on mortality.

**Conclusions:**

Our study strengthens the evidence of adverse heat effects on respiratory mortality in Northern Europe by identifying vulnerable subgroups and subregions. This may contribute to the development of targeted policies for adaptation to climate change.

**What this study adds**

This work is exploring the heat effects on respiratory mortality in Central-Northern Europe, and the effect modification by area-level characteristics. The analysis was conducted at a small-area level within Norway, Germany, and England and Wales, covering the whole country, including both urban and non- urban settings. This level of analysis resulted in better exposure characterization and, consequently, in more accurate effect estimates. The investigation of the area-level potential effect modifiers contributes to the limited evidence on the modification of the heat effects on cause-specific mortality, as most of the published studies have focused on all-cause mortality.

## Introduction

Respiratory diseases are the third leading cause of death in Europe, causing some 364,000 deaths in 2019 (8% of all deaths), following circulatory diseases and cancer mortality. The majority of respiratory deaths occur among the elderly and account for 7% of all deaths among women and 9% among men.^1^

Previous findings have provided substantial evidence that high temperatures are associated with effects on all-cause and cause-specific mortality,^2–11^ a very critical issue under climate change. The association between daily mortality and temperature across the year, on the same or a few preceding days, exhibits a U-shape.^3,4,6,12,13^ There is an optimum temperature value (“threshold”) at which minimum mortality is observed, while mortality increases with temperatures lower or higher than this point. Warmer locations have higher thresholds than colder ones, probably as a result of the population’s adaptation to the local climate, either physiologically or driven by differential exposure patterns determined by cultural or social characteristics.^3,12,14^

Several factors may modify the impact of elevated temperatures on health, including individual and area-level characteristics. Previous research has indicated population subgroups that are most vulnerable to heat.^9,15,16^ A recent systematic review on the modification of the temperature effects on mortality^16^ found strong evidence of higher heat mortality risks among the elderly and women, while the evidence of effect modification for other individual-level factors such as education, occupation, race, marital status, and chronic conditions was limited or suggestive.^16^

Evidence is even less consistent regarding area-level characteristics. Evidence for effect modification by community-level socioeconomic status (SES), type of area (urban/rural), green areas, use of air conditioning, local climatic conditions, and previous winter mortality is limited or suggestive.^16^ Weak evidence was reported for effect modification by population density, healthcare facilities, proximity to water, housing quality, and air pollution.^16^ However, most of the included studies examined effect modification by individual characteristics and all-cause mortality; hence, further research is needed to investigate patterns by area-level factors,^16^ especially so for cause-specific mortality.

The majority of studies on the heat effects on mortality are time-series studies of city-wide data^3,5^ and few have examined the differentiation of the effects within an urban area.^10,17^ A smaller spatial level of analysis can provide more accurate exposure estimates. The investigation of the temperature effects on mortality at a small-area level and with broad geographical coverage is of great importance as it could reveal exposure and/or risk contrasts between areas, that could potentially be explained by differential population or geographical characteristics.^18^

Under climate change and global warming, air temperature is expected to increase with severe public health impacts. For the first time in 2019, the Global Burden of Diseases, Injuries, and Risk Factors Study,^19^ added the non-optimal temperatures as risk factors. According to the 2022 Global Report of the Lancet Countdown,^20^ a 68% increase in heat-related deaths was observed between 2000–2004 and 2017–2021, while vulnerable populations were exposed to 3.7 billion more heatwave days in 2021 than annually in 1986–2005.

The EU-Horizon2020 project “Exposure to heat and air pollution in Europe–cardiopulmonary impacts and benefits of mitigation and adaptation” (EXHAUSTION)^21^ aims to contribute to advancing knowledge and methods regarding the temperature-health link by investigating the heat effects on cardiopulmonary mortality and morbidity and assessing effect modifiers. In the present work, we investigated the short-term effects of heat on respiratory mortality during the warm season studied at a small administrative area level and their modification at a nomenclature of territorial units for statistics (NUTS)-3 area level by several area-level characteristics in Norway, England and Wales, and Germany, in the framework of the EXHAUSTION project. The focus on respiratory mortality was driven by the distinct pathophysiological mechanisms triggered by heat exposure,^22^ in comparison to those associated with cardiovascular mortality and potentially varying effect modification patterns.

## Data and methods

### Data

We collected data at a small-area level on daily counts of respiratory deaths (International Classification of Diseases [ICD]-9: 460-519; ICD-10: J00-J99), daily mean air temperature (^o^C), and area-level characteristics from Norway, England and Wales, and Germany. The time scale and the area level of the aggregated data slightly differed by country. Small-area mortality data were available for Norway at municipality-level, a subdivision of the larger area of district, for 1996–2018 from the Cause of Death Registry of Norway; for England and Wales, at the Lower Super Output Area (LSOA) level, a subdivision of Local Authority Districts (LAD), for 2000–2018 from the Office of National Statistics (ONS); for Germany, at district level for 2000–2016 obtained from the German FDZ, Research Data Center. Data were also available for those aged above 65 years, and, for Norway and Germany, by sex.

Data on daily mean air temperature at a spatial resolution of 1 km × 1 km were available for the different countries for the corresponding time periods. In Norway, a spatio-temporal observational gridded dataset was used, accounting for temperature and precipitation. In England and Wales, a corresponding dataset was also used, based on inverse-distance weighted regression of measurements from weather stations, including predictors based on altitude, coastal influence, and urban density. In Germany, a hybrid spatio-temporal regression-based model was applied, accounting for satellite-based land surface temperature, elevation, normalized difference vegetation index, percentages of the urban fabric, arable land, pastures, forests, and inland waters. The data were aggregated at the same spatial level as the mortality data (eAppendix A1 and eTable 1; http://links.lww.com/EE/A237).

For the identification of area-level characteristics that may modify the heat-mortality relationship, data on socio-economic and land use characteristics were obtained at the NUTS-3 level, which may differ (depending on the country) from the small-area level underlying the main analysis. The NUTS classification is a hierarchical system for dividing up the economic territory of the EU and the UK for the purpose of the collection, development, and harmonization of European regional statistics, socio-economic analyses of the regions, and framing of EU regional policies. Specifically, NUTS-3 areas are regions with 150,000–800,000 inhabitants. For practical reasons, the NUTS classification generally mirrors the Member States’ territorial administrative division, which supports the availability of data and the implementation capacity of policies.^23^

We chose the NUTS-3 level as we obtained the potential effect modifiers at a uniformly defined area level for all areas from EUROSTAT.^24^ Further, regions within the same NUTS-3 area are assumed to be relatively homogenous regarding socio-economic and land use characteristics. We used the average values of the selected variables for each area’s available years within the study period. We obtained data on: population density (number of inhabitants per square kilometer [km2]); percentage of the population over 65 years; ratio of employed population and population aged 20–64 (for England and Wales we used data on population aged 16–64 due to availability); Gross Domestic Product (GDP) per inhabitant (Euro [€]); urban type; mountain type; coastal type (eTable 2; http://links.lww.com/EE/A237).

For England and Wales, data on employment and GDP were obtained from the Office of National Statistics^25^ as they were not available in Eurostat. GDP was available in British Pounds and was converted to Euros (1.00 British Pound = 1.18 Euros as on 18 July 2022). In some NUTS-3 areas, the employed population was higher than the population aged 20–64 (16–64 for England and Wales), which may be due to the commuting of people employed in an area who actually reside in another one.

Land use characteristics were obtained from CORINE land cover for 2012,^26^ a representative year in the middle of the study period, and included the percentage of urbanized area and the green area in square kilometers per 100,000 persons per NUTS-3 area (eAppendix A2; http://links.lww.com/EE/A237).

Finally, data on the average concentrations of particulate matter with an aerodynamic diameter <2.5 μm (PM_2.5_) were used as possible modifiers of the heat effects. Daily data, estimated by area-specific exposure assessment methods at 1 km × 1 km resolution, were averaged over the study period by NUTS-3 area (eAppendix A3; http://links.lww.com/EE/A237).

### Statistical Methods

We applied a multi-stage analysis to investigate the temperature-respiratory mortality association in the warm period of the year, defined as the 5-month period May–September. Data at a small-area level were analyzed separately for each country following a harmonized protocol, and subsequently, the results were pooled at the country level and overall.

Initially, we created large area-specific datasets (district-specific for Norway and Germany, LAD-specific for England and Wales) by stacking together the time series of all the small areas (municipalities for Norway, districts for Germany, LSOAs for England and Wales) belonging to the same large one. For each large-area dataset^27,28^ we analyzed the small-area time series via Poisson regression allowing for overdispersion and used distributed lag non-linear models^29^ to describe the temperature-mortality association in the warm period as:


$$\begin{array}{l} log\left[E\left(Y_{ij}\right)\right]=\alpha +crossbasis\left(Tmean_{0-1}\right)\;\\ \;+small\;area_{j}*year*month*dow, \end{array}$$

where: $Y_{ij}$ is the number of deaths in the day *i* in small area *j*; $Tmean_{0-1}$ is the daily mean temperature, fitted in the cross-basis term composed of a B-spline for the exposure-response function with one internal knot (50th percentile of the small-area-specific distribution) and $small\;area_{j}*year*month*dow$ is the 4-way interaction between small areas, year, month and day of the week to account for seasonality adjustment, resembling a case-crossover design with time trend adjustment achieved separately for each small area. For Norway and England and Wales these were pooled to produce the best linear unbiased prediction (BLUP) estimates at large area scales, while for Germany, as only one geographical scale was available, the interaction term only included year, month, and day of the week. The lag period was restricted to 2 days (lag 0–1) to focus on the short-term effect of heat. We tested the sensitivity of our findings by (1) using lags 0–3 for the temperature and (2) restricting the warm period to the 3 warmest months (June–August).

In the second stage, we applied a multivariate meta-analysis to pool the first-stage estimates and derive the best linear unbiased prediction of the overall cumulative exposure-response association separately for each country.^30^ Main results are reported as relative risks (RR), with corresponding 95% confidence intervals (95% CI), for increases in daily mean temperature from the 75th to the 99th percentile of the area-specific distributions. As a sensitivity analysis, we estimated the RR relative to the Minimum Mortality Temperature (MMT) instead of the 75th percentile. Results are reported also for people over 65 years and by sex.

Finally, we pooled the RRs estimated from the second stage, to obtain a pooled estimate by random effects meta-analysis.

### Effect modification by vulnerability factors or other potential effect modifiers

The investigation of effect modification of the temperature-respiratory mortality association was conducted at the NUTS-3 area level to have a harmonized list of modifiers provided by the same source, as outlined in the data section. For Norway, the level of the first-stage effect estimates was the same as the NUTS-3 level; for England and Wales, a random-effect multivariate meta-analysis was applied to obtain the exposure-response functions at the NUTS-3 level, pooling the LAD-specific effect estimates from the first stage belonging to the same NUTS-3 area; for Germany, as the level of the first stage estimates (district level) covered several NUTS-3 areas in some cases (and was exactly the same as NUTS-3 level for the rest of them), the data on potential effect modifiers were aggregated at the district level, if necessary.

We applied multivariate meta-regression models, with a maximum likelihood estimator for the between-studies variance, to include vulnerability factors and potential effect modifiers as covariates, to explain part of the heterogeneity of the temperature-mortality association between the NUTS-3 areas.^31^ As dependent variable we used the NUTS-3 area-specific coefficients of the exposure-response function, as explained above.

The multilevel random-effects model included random intercepts for NUTS-3 areas nested within countries. As several of the examined factors are related to urbanicity and population density, we adjusted for population density in the models for the covariates that had an absolute value of the Spearman correlation coefficient with population density less than 0.7 and when assessing the effect of the coastal and mountain type of the region. We did not adjust for population density when assessing urbanicity, as its definition is based on population density.

Results are reported as RRs, with corresponding 95% CI, for increases in daily mean temperature from the 75th to the 99th percentile at the 5th and 95th percentile of the distribution of each effect modifier. As a sensitivity analysis, we estimated the corresponding RRs relative to the MMT instead of the 75th percentile. The statistical significance of the effect modifiers was assessed using a Wald Test. All analyses were carried out using R software (version 4.2.1) with gnm, dlnm, and mixmeta package.

## Results

### Heat effects on respiratory mortality

**Figure 1.**
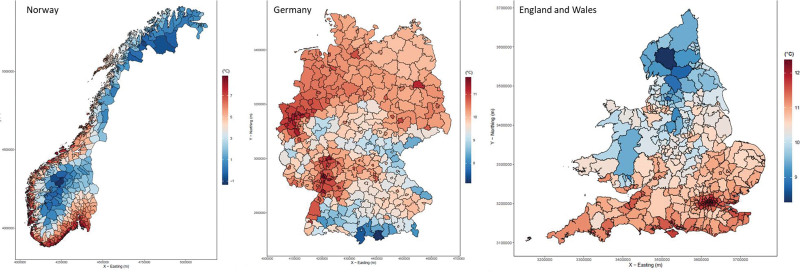
Annual mean air temperature by small area in Norway, Germany, and England & Wales, year 2015

The heat effects on respiratory mortality were estimated in 346 areas in England and Wales, 357 in Norway, and 380 in Germany, analyzing data from a total of 35,490 small areas (Table 1). The mean temperature was 10.54 °C in Norway, 15.34 °C in England and Wales and 15.84 °C in Germany (Table 1). The country-specific mean temperature distribution of 2015 as indicative year is presented in Figure 1. The respiratory mortality rate was 642 deaths per 100,000 inhabitants in Norway, 425 deaths in England and Wales, and 327 deaths in Germany (Table 1).

**Table 1.** Descriptive statistics for temperature and mortality data of the warm season of the study period

			Population		Respiratory mortality			Air temperature (^o^C)		
	Number of small areas (large areas)	Time period	Million inhabitants (2016)	Total Number events	Daily deaths at small area- level mean (SD)	Mortality rate (per 100,000 inhabitants)	Mean (SD)	MMT	75th	99th
Norway	357 (11)	1996–2018	5.21	33,468	0.03 (0.18)	642	10.54 (4.51)	−5.90	13.68	20.23
England and Wales	34,753 (346)	2000–2018	58.38	247,836	0.00 (0.10)	425	15.34 (3.11)	13.70	17.36	22.70
Germany	380 (380)	2000–2016	82.52	299,249	0.30 (0.68)	327	15.84 (3.92)	10.15	18.43	24.89

**Figure 2.**
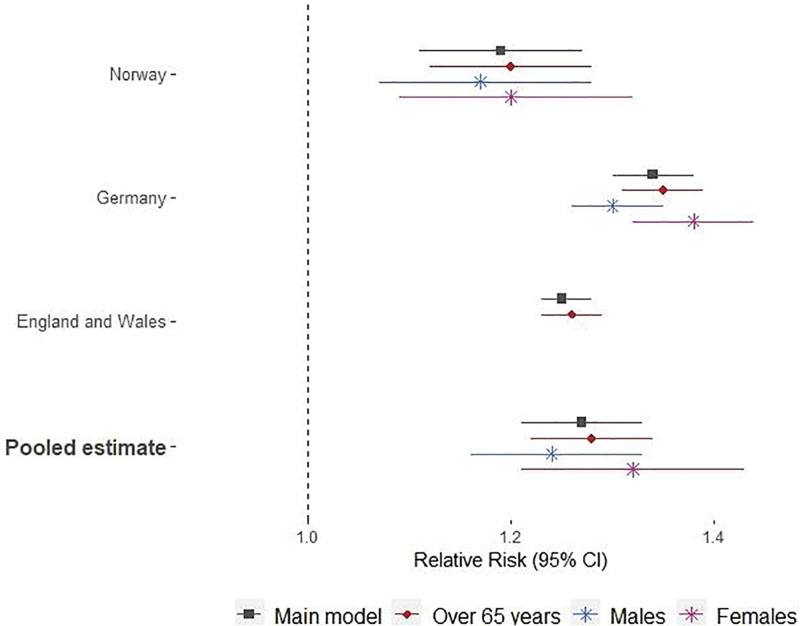
Relative Risks (and 95% confidence intervals) per increase in the 2-day mean temperature from the 75th to the 99th percentile of the area-specific distribution (main model) and corresponding results from age- and sex-specific sensitivity analyses by country.

The pooled RR of respiratory mortality for mean temperature increases from the 75th to the 99th percentile was 1.27 (95% CI = 1.19, 1.34), with higher effects in Germany (Figure 1 and eTable 3; http://links.lww.com/EE/A237). Slightly higher risks were observed among the elderly (65+ years; RR = 1.28; 95% CI = 1.20, 1.35) and females (RR = 1.30; 95% CI = 1.13, 1.49) (Figure 2). Sensitivity analyses showed comparable results when restricting the analysis to the 3 warmest months, while higher effects were identified when a lag of 0–3 or a wider temperature increment was considered (starting from MMT). The difference in RRs, when the MMT was used, was more pronounced in Norway, where the MMT was estimated to be at the minimum temperature value (eTable 3; http://links.lww.com/EE/A237). Country-specific curves are reported in the eAppendix (eFigure 1; http://links.lww.com/EE/A237).

### Effect modification by vulnerability factors or other potential effect modifiers

The effect modification of the heat effects on respiratory mortality was assessed in 536 NUTS-3 areas (145 in England and Wales, 380 in Germany, and 11 in Norway). Descriptive statistics and correlations among the effect modifiers are reported in Table 2, and in the eAppendix (eTables 4–9; http://links.lww.com/EE/A237).

**Table 2.** Mean values of selected effect modifiers variables for all the available years within the study period

	Vulnerability factor/Potential effect modifier	5th perc	25th perc	Median	75th perc	95th perc
**Socio-economic characteristics**	Population density (inhabitants per km^2^)	65.00	127.00	271.00	947.00	3942.00
	Urbanized area (%)	3.00	5.00	8.00	20.00	66.00
	Green area per 100.000 inhabitants	4.37	71.13	323.85	714.75	1447.14
	Population aged over 65 years (%)	13.36	18.51	20.16	21.72	24.68
	Gross domestic product at current market prices (€)	17570.59	21594.12	25889.45	32476.47	54541.18
	Ratio of employed population and population aged 20–64 (%)	56.40	70.55	77.11	83.97	122.50
**Air pollution**	PM_2.5_ (μg/m^3^)	9.23	11.3	12.01	13.35	15.57
**Region type (%**)	Urban type					
	Predominantly urban regions				38.14%	
	Intermediate regions				40.04%	
	Predominantly rural regions				21.82%	
	Mountain type					
	>50% of population live in mountain areas				0.38%	
	>50% of surface in mountain area				4.70%	
	>50% of population lives and 50% of surface in mountain areas				6.20%	
	Non-mountain region				88.72%	
	Coastal type					
	Sea border				19.29%	
	>50% of the population within 50 km of coastline				9.74%	
	Non-coastal				70.97%	

**Figure 3.**
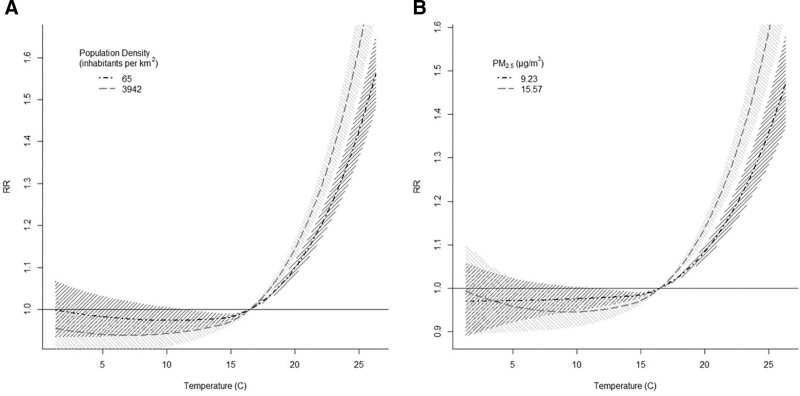
Predicted temperature-respiratory mortality association during the warm period for at the low and high levels (5th and 95th percentile, respectively) of (A) population density and (B) PM_2.5_ concentrations.

When investigating one modifier at a time, heat effects were increased in more dense areas compared to less dense ones, with the corresponding increases in mortality being 32% (95% CI = 28%, 37%) and 22% (95% CI = 19%, 26%), respectively (Table 3 and Figure 3). Similar results were estimated for more urbanized areas compared to less urbanized ones. Predominantly urban regions had the highest heat effects on mortality, while intermediate and predominantly rural regions had lower and comparable heat effects (Table 3). Reversely, lower heat-related mortality was observed in areas with higher levels of green compared to those with lower levels, although the differences were not pronounced (Table 3). Results were inconsistent for mountain and coastal types, even when adjusted for population density (Table 3).

**Table 3.** Relative risks (and 95% CIs) for the effect on respiratory mortality for an increase in temperature from the 75th (16.5 ^O^C) to the 99th percentile (22.4 ^O^C) at the low and high level of the effect modifier (5th and 95th percentile respectively)

Effect modifier	5^th^ percentile	RR (95 % CI)	
	95^th^ percentile		
		Unadjusted models	Adjusted for population density
Population density (inhabitants per km^2^)	65.00	1.22 (1.19, 1.26)*	
	3942.00	1.32 (1.28, 1.37)*	
Urbanized area (%)	3.00	1.22 (1.19, 1.26)*	
	66.00	1.34 (1.28, 1.39)*	
Green area per 100,000 inhabitants	4.37	1.27 (1.24, 1.29)	
	1447.14	1.24 (1.20, 1.27)	
Gross domestic product at current market prices (€)	17570.59	1.24 (1.22, 1.27)	1.23 (1.20, 1.26)
	54541.18	1.28 (1.24, 1.31)	1.23 (1.19, 1.27)
Ratio of employed population and population aged 20–64 (%)	56.40	1.23 (1.19, 1.26)	1.21 (1.17, 1.25)
	122.50	1.32 (1.26, 1.39)	1.28 (1.21, 1.35)
Population aged over 65 years (%)	13.36	1.30 (1.25, 1.35)*	1.23 (1.17, 1.29)
	24.68	1.20 (1.16, 1.25)*	1.23 (1.18, 1.28)
PM_2.5_ (μg/m^3^)	9.23	1.18 (1.15, 1.22)*	1.19 (1.15, 1.23)*
	15.57	1.34 (1.30, 1.38)*	1.31 (1.26, 1.36)*
	Category		
Urban type	Predominantly urban regions	1.29 (1.25, 1.33)*	
	Intermediate regions	1.20 (1.16, 1.25)*	
	Predominantly rural regions	1.23 (1.17, 1.30)*	
Mountain type	>50% of population live in mountain areas	1.45 (1.05, 2.01)	1.42 (1.03, 1.97)
	>50% of surface in mountain area	1.25 (1.15, 1.36)	1.26 (1.16, 1.36)
	>50% of population live and 50% of surface in mountain areas	1.15 (1.05, 1.25)	1.11 (1.02, 1.21)
	Non-mountain region	1.26 (1.24, 1.29)	1.23 (1.20, 1.27)
Coastal type	Sea border	1.25 (1.21, 1.29)	1.22 (1.17, 1.27)
	>50% of the population within 50 km of coastline	1.29 (1.23, 1.34)	1.26 (1.20, 1.32)
	Non-coastal	1.25 (1.22, 1.28)	1.23 (1.19, 1.27)

* Statistically significant effect modifier at 5% level of the heat-mortality association based on the Wald test.

In areas with high GDP per inhabitant and a high ratio between employed population and population aged 20–64, the heat effect on mortality was slightly higher than in areas with lower levels of these variables, but the difference was not statistically significant (Table 3). Adjusting for population density, the differences in heat effects between the levels of the modifiers were even smaller.

Areas with a high percentage of people over 65 years were estimated to have statistically significant lower heat effects on mortality compared to areas with a lower percentage of this age group. When we adjusted for population density, the percentage of elderly no longer modified the heat-mortality association, with similar increases in mortality for areas with a high and low proportion of elderly (Table 3).

The only statistically significant modifier of the heat-mortality association after adjustment for population density was PM_2.5_. In areas with high levels of PM_2.5_, the estimated increase in heat-related mortality was estimated to be 31% (95% CI = 26%, 36%), while the corresponding increase in areas with low levels was 19% (95% CI = 15%, 23%), after population density adjustment (Table 3 and Figure 3). Relative risks for temperature increase from the MMT to the 99th percentile were of the same magnitude as the RRs of the main analysis (eTable 10; http://links.lww.com/EE/A237). Results by country are also presented in the eAppendix (eTable 11; http://links.lww.com/EE/A237).

## Discussion

Our study is among the few assessing the heat effects on respiratory mortality at small administrative area level for large regions across Europe during the warm period and factors that might modify this association. Increased mortality risk during heat exposure was estimated for all areas, with females being more vulnerable. A high degree of urbanicity and higher PM_2.5_ levels were associated with stronger heat effects.

Exposure to heat mainly affects patients with chronic respiratory diseases. More specifically, thermoregulatory responses to heat stress, particularly those involving the respiratory system, may be ineffective at dissipating excess heat, thus increasing their risk of developing heat stress conditions such as dehydration and heatstroke.^32^ Alterations in blood composition towards a procoagulant condition might increase their risk of developing pulmonary vascular resistance, and these modifications in the blood vessels may initiate a respiratory distress syndrome by activating the complement system.^22^ Furthermore, the respiratory system is the main target of gases, aerosols, and particles present in air pollution. Heat can exacerbate air pollution, especially in urban areas, leading to the formation of ground-level ozone and other pollutants. Ozone provokes oxidative damage to the respiratory epithelium, resulting in lung inflammation, decrements in lung function, worsening of respiratory tract symptoms, and increased airway reactivity. Further, particulate matter, via enhanced oxidative stress, may disrupt lung endothelial cell barrier integrity, thereby inducing lung dysfunction and adverse cardiopulmonary outcomes.^32^ These specific pathophysiological mechanisms for respiratory mortality triggered by heat exposure, along with cause–specific effect modification patterns, can lead to the identification of vulnerable populations to be targeted by public health authorities.

Our findings support previous results for the adverse heat effects on respiratory mortality. A large meta-analysis including 26 studies around the world^33^ found a 2.32% (95% CI = 2.02, 2.62) increase in respiratory mortality per 1 °C increase in temperature above the threshold. Focusing on a similar study area as in our analysis, a study in eight European north-continental cities, found a 6.10% (95% CI = 2.46, 11.08) increase in respiratory mortality per 1 °C increase in maximum apparent temperature above the city-specific threshold.^3^ In England and Wales^5^ the increase in respiratory mortality was 4.10% (95% CI = 3.50, 4.80) for a corresponding temperature increase, while the increase in mortality associated with an increase from the 90th to the 99th temperature percentile was 23.30% (95% CI = 13.60, 33.80) in three German cities.^4^ Previous studies have proposed that women and the elderly are more vulnerable to the heat effects.^3,27,34–36^ However, further investigation of the heat effects’ differentiation by sex in specific age groups is needed, as a possible different sex distribution in vulnerable age groups can lead to misleading conclusions regarding sex-specific effects.

Few studies have investigated the heat effects on mortality in rural areas. A meta-analysis on heat-related mortality risk among rural populations^37^ estimated an increase of 3.00% (95% CI = 1.30, 4.80) in all-cause mortality per 1 °C increase in daily mean temperature, suggesting that the excess mortality risks in rural areas are of similar magnitude to those in urban ones. A recent study by Gasparrini et al.^27^ assessed the heat effects in England and Wales at a small-area level and reported higher mortality rates in urban areas and a significant mortality burden in non-urban areas. However, further evaluation of the heat effects on mortality in rural populations is needed. Our findings from the analysis by type of region support that the health burden on respiratory mortality is considerable in rural areas, where the percent change in heat-related mortality was over 20%.

The potential effect modification by area-level characteristics has not been extensively investigated and has been investigated mostly within urban areas. Several studies have identified that areas with high population density and coverage of buildings are more affected by heat.^10,36,38,39^ Our results are consistent with previous assessments, although our analysis was not limited to urban settings. Maybe this could be explained as in urban environments, which are characterized by high density of population and buildings, there is increased heat storage from built surfaces, decreased latent and sensible heat exchange (and thus reduction of evaporative cooling), and heat added by human activities. All these factors modulate urban local climates and form “Urban Heat Islands” (UHI) that make cities hotter than surrounding non-urban environments, enhancing the heat-related effects on human health.^40^

Higher presence of green has been associated with smaller heat effects on mortality,^36,38,39,41–43^ which is in agreement with our findings, although the differences were not pronounced. However, as already discussed, heat effects are lower in non-urban areas (rural and intermediate areas), which have a higher presence of green areas, compared to urban areas (the median was 892.40 km2 per 100,000 persons in rural areas and 455.25 km2 per 100,000 persons in intermediate areas of our study area, compared to 55.13 km2 per 100,000 persons in urban areas). Vegetation can contribute to a reduction of heat exposure, as it is able to absorb direct solar radiation, change the albedo of background surfaces, and have a cooling effect through evapotranspiration.^44^ Also, we found that elevated average levels of PM_2.5_ are associated with a higher increase in heat-related mortality, which is supported by a large multi-country study.^38^ This could be explained by the underlying mechanisms that particulate matter affects the respiratory system, via enhanced oxidative stress,^32^ as mentioned above.

Evidence is contradictory when considering SES indicators as effect modifiers for the heat-mortality association. Higher effects were observed in areas with a large percentage of manual workers,^39^ and in areas with higher poverty and more residents without a high school degree,^36^ as indices of low socioeconomic status. Also, higher heat effects have been reported in areas with a higher unemployment rate.^10^ Gasparrini et al.^27^ reported an increased risk in areas with greater socioeconomic deprivation but without pronounced differences between the different socioeconomic groups. Liu et al.^45^ observed significant heat effects on mortality only in lower SES areas in Hong Kong, while Murage et al.^42^ did not identify SES as a statistically significant modifier of the association in London.

Our findings point to areas with higher GDP per capita, considering it as an indicator of high SES, having higher heat effects on mortality. This is in accordance with the results from a multi-country analysis^38^ supporting that GDP and educational level are positively associated with heat-related mortality. A possible explanation might be an association between economic development with features of urbanization such as the UHI.^38^ This also can be supported by our findings as when we adjust for population density, an indicator of economic development and urbanization, GDP does not seem to be associated with the magnitude of the heat effects. However, further studies, including individual-level socio-economic indicators and indices of social and economic inequalities, are needed.

Although age has been identified as a vulnerability factor of heat-related outcomes, the age distribution of area-specific populations may affect the vulnerability to heat in a different way. When previously the proportion of elderly has been assessed as a city-level modifier, no differences in heat-related mortality were observed between areas.^38,39,43^ In our study, small areas with a higher proportion of elderly displayed a lower heat effect on respiratory mortality that did not persist following adjustment for population density to account for the varying urbanicity degree. The latter is in accordance with the main analysis results that displayed a very small difference in effects between all ages and the elderly. As during the years in our study heat warning systems have been available, the elderly, as a subgroup vulnerable to heat, may have complied and taken precautions to avoid exposure. Further, the stronger effects observed in areas with a lower proportion of elderly, when not accounting for population density, may reflect the stronger heat effects that are experienced in urban centers which are traditionally more densely populated and record a lower proportion of elders compared with rural ones.^46^

Our study strengthens the limited evidence in Europe on respiratory mortality heat effects using high-resolution exposure data assigned at small-area level spanning over three European countries. Most studies on temperature–mortality association were based on city-wide time-series data, while effect modification has been assessed between cities or urban areas. We obtained harmonized data on potential effect modifiers from a common database, avoiding differences or misclassifications induced by varying definitions across areas. Another strength of our study is that the applied meta-analytic models take into account the multivariate structure of the estimated non-linear associations, and the multivariate meta-regressions included specific predictors to examine possible effect modification. The applied extended two-stage design, adopted from Sera and Gasparrini,^31^ provides a useful and more flexible tool for modeling complex risks associated with environmental factors compared to the classical two-stage approach.

A limitation of our study is that the high-resolution temperature data assigned at a small-area level were produced by different approaches in each country, using different predictors and different sources for temperature observations. The same holds for the air pollution levels used as an effect modifier. Another limitation is the measurement error induced by the lack of information on the mobility of the population. This is an inherent limitation when using population time series as data on time-location-activity patterns are not available and could potentially be addressed by future research.

In conclusion, our study indicates a risk of heat-related mortality in Central-North European areas. Women are more vulnerable to heat, and people that live in dense areas characterized by higher PM_2.5_ concentrations. Identifying the main drivers of vulnerability to the effects of heat is of great importance as it helps policy makers and public health authorities in developing more targeted climate change adaptation measures.

## Supplementary Material


