# Supplementary material for: Assessing heat effects on respiratory mortality and location characteristics as modifiers of heat effects at a small area scale in Central-Northern Europe
Source: Environ Epidemiol. 2023 Sep 13;7(5):e269. doi: 10.1097/EE9.0000000000000269 (PMC10569755; doi:10.1097/EE9.0000000000000269)
Supplement: Supplementary file 1 [file ee9-7-e269-s001.docx]

**eAppendix**

**A1. Air temperature data**

1. **Norway**

Spatial-temporal temperature model: Daily mean air temperatures on a 1 km grid across Norway for the years 1995 to 2018 are obtained from the seNorge2 dataset. On the Norwegian mainland, seNorge2 provides high-resolution fields of daily temperature (mean, min and max); it is updated daily and presented on a high-resolution grid (1 km of grid spacing). The climate archive goes back to 1957. The spatial interpolation scheme builds upon classical methods, such as optimal interpolation and successive-correction schemes. The dataset is continuously updated based on measurement data.

1. **England and Wales**

Daily mean air temperatures for the years 2000 to 2018 were extracted from the daily 1x1km gridded daily HadUK-Grid data from Met Office. The grid Is available at the CEDA repository https://archive.ceda.ac.uk/). Extracted time series at each grid point were then averaged daily by LSOA with weights proportional to the overlap between the grid square and the LSOA boundary.

1. **Germany**

Satellite-based spatial-temporal temperature models: Daily mean air temperatures on a 1 km grid across Germany for the years 2000 to 2016 were estimated by a 3-stage regression-based modelling approach . The model incorporated satellite-based land surface temperature (LST) data, ground-based air temperature (Ta) measurements, and spatial predictors, including NDVI, percent urban, and elevation. In the first stage, for grid/day combinations with both Ta and LST data available, Ta measurements were regressed against LST by a linear mixed-effects model including day-specific random intercepts, fixed and random slopes for LST, and spatial predictors. In the second stage, the first-stage model was used to predict Ta in grid cells without Ta measurements but with daily LST data from satellite. Because LST data are not available in all grid/day combinations due to cloud, snow, or measurement failures, in the third stage, we fitted a generalized additive mixed model with thin plate splines for spatial smoothing. This model was applied to estimate Ta when LST data were not available by using the association of predicted Ta values in a certain grid cell with the average Ta values of surrounding measurement stations within a buffer of 60 km from the grid cell centroid. Thus, fully spatiotemporally covered predictions of Ta were obtained after the third stage.

**A2. Definition of the land use characteristics**

For the definition of the urban areas, we used the following classes from the CORINE Land Cover (CLC) nomenclature:

**Class 1: Artificial areas**

**Class 1.1 Urban fabric (includes 111, 112)**

Areas mainly occupied by dwellings and buildings used by administrative/public utilities, including their connected areas (associated lands, approach road network, parking lots). The continuous urban fabric class is assigned when urban structures and transport networks are dominating the surface area. > 80% of the land surface is covered by impermeable features like buildings, roads and artificially surfaced areas. Non-linear areas of vegetation and bare soil are exceptional. The discontinuous urban fabric class is assigned when urban structures and transport networks associated with vegetated areas and bare surfaces are present and occupy significant surfaces in a discontinuous spatial pattern. The impermeable features like buildings, roads and artificially surfaced areas range from 30 to 80 % land coverage.

For the definition of the green areas, we used the following classes from the CORINE Land Cover (CLC) nomenclature ( Keijzer et al. 2019 de Keijzer C, Tonne C, Sabia S, Basagaña X, Valentín A, Singh-Manoux A, Antó JM, Alonso J, Nieuwenhuijsen MJ, Sunyer J, Dadvand P. Green and blue spaces and physical functioning in older adults: Longitudinal analyses of the Whitehall II study. Environ Int. 2019 Jan;122:346-356. doi: 10.1016/j.envint.2018.11.046. Epub 2018 Nov 29. PMID: 30503316; PMCID: PMC6571017.)

**Class 1: Artificial areas**

**Class 1.4 Artificial non-agricultural vegetated areas**

**141 Green urban areas**

Areas with vegetation within or partly embraced by urban fabric. This class is assigned for urban greenery, which usually has recreational or ornamental character and is usually accessible for the public.

**Class 2: Agricultural areas**

**Class 2.1 Arable land (includes 211, 212, 213)**

Lands under a rotation system used for annually harvested plants and fallow lands, which are rain-fed or irrigated. Includes flooded crops such as rice fields and other inundated croplands.

**Class 2.2 Permanent crops (includes 221, 222, 223)**

All surfaces occupied by permanent crops, not under a rotation system. Includes ligneous crops of standards cultures for fruit production such as extensive fruit orchards, olive groves, chestnut groves, walnut groves shrub orchards such as vineyards and some specific low-system orchard plantation, espaliers and climbers.

**Class 2.3 Pastures (includes 231)**

Lands that are permanently used (at least 5 years) for fodder production. Includes natural or sown herbaceous species, unimproved or lightly improved meadows and grazed or mechanically harvested meadows. Regular agriculture impact influences the natural development of natural herbaceous species composition.

**Class 2.4 Heterogeneous agricultural areas (includes 241, 242, 243,244)**

Areas of annual crops associated with permanent crops on the same parcel, annual crops cultivated under forest trees, areas of annual crops, meadows and/or permanent crops which are juxtaposed, landscapes in which crops and pastures are intimately mixed with natural vegetation or natural areas.

**Class 3: Forest and semi-natural areas**

**Class 3.1 Forests (includes 311, 312, 313)**

Areas occupied by forests and woodlands with a vegetation pattern composed of na-tive or exotic coniferous and/or broad-leaved trees and which can be used for the pro-duction of timber or other forest products. The forest trees are under normal climatic conditions higher than 5 m with a canopy closure of 30 % at least. In case of young plantation, the minimum cut-off-point is 500 subjects by ha

**Class 3.2 Shrubs and/or herbaceous vegetation associations (includes 321, 322, 323, 324)**

- Temperate shrubby areas with Atlantic and Alpine heaths, sub -Alpine bush and tall herb communities, deciduous forest recolonisation, hedgerows, dwarf conifers.
- All transitional forest development stages (regenerative and degenerative: natural development of forest – bushy formations on abandoned meadows, pastures or forest clear cut and also forest after calamities of various origin) should be classified as 324.
- Shrubby formation with sparse trees (< 15 % canopy closure in climax stage and the height of trees can be > 5-7 m) composed of dwarf forms of *Betula spp.* and *Salix spp.,* plus *Vaccinium spp. Empetrum migrum, Ledum palustre, Carex spp., Cladonia spp.,* etc. (cover > 50 % of sur-face) frequently interrupted by rock outcrops (typical of Scandinavia and the Northern Atlantic) should be classified as **moors and heathland** – tundra (322).
- Mediterranean and sub-Mediterranean evergreen sclerophyllous bush and scrub (maquis, gar-rigue, matorral, phrygana sensu lato), re-colonisation and degradation stages of broad-leaved evergreen forests.
- Dry thermophilous grasslands of the lowlands, hills and mountain zone. Poor Atlantic a sub-Atlantic mat-grasslands of acid soils; grasslands of decalcified sands; Alpine and sub Alpine grasslands. Humid grasslands and tall herb communities; lowland and mountain mesophile pas-tures and hay meadows.

**Α3. Air pollution data**

1. **Norway**

a. UBM Model: Daily mean concentrations of PM2.5, O3, and NO2 for 2000-2015 at a 1 km resolution will be obtained using the DEHM-UBM model setup, which combines the Danish Eulerian Hemispheric Model (DEHM)] with the Urban Background Model (UBM). The geographic domain of DEHM covers the Northern hemisphere, while UBM covers a limited area of interest with a high spatial resolution (1 km x 1 km). Full non-linear chemistry is included in DEHM, while the NO-NO2-O3 chemistry is included in UBM. This combination makes it possible to describe the long-range atmospheric transport of pollutants into and within Europe, while still having a high spatial resolution in the output over e.g. populated areas. In the current setup, a new high-resolution emission inventory for the Nordic region has been used as input to the UBM model, while the emissions applied in DEHM is based on the EMEP emission database.

1. **England and Wales**

Satellite-based spatial-temporal air pollution models: Daily mean concentrations of PM10 (2008-2018) and PM2.5 (2008-2018) were obtained for each 1-km grid cell of England and Wales by a multi-stage approach. Specifically, a first stage was applied to extend the number of monitoring stations measuring PM2.5, by using co-located PM10 data. Concurrently, a second stage was used to fill in satellite-AOD missing data using modelled AOD at 3-hr resolution and multiple bands from CAMS as main predictors. Then, stage 3 was aimed at calibrating monitored PM data to satellite AOD, meteorological parameters, dispersion model outputs, and multiple spatial parameters (modelled PM2.5 from chemical transport models, meteorological variables, population density, light-at-night, road network density, elevation etc.). For this, a machine learning methodology, the random forest, has been applied. Finally, in stage 4 the model trained in the previous step has been used to predict daily mean PM10 and PM2.5 in all grid cells (1-km) and days of the aforementioned domain. The so-derived exposures at fine grid have been reported at the LSOA level, by averaging all the intersection grids of the LSOA, with grid cells weighting proportionally to the intersection area (grids totally intersecting weighted 1, those only partially intersecting weighted a fraction of 1.

1. **Germany**

Optimal Interpolation (chemical REM-CALGRID model): Air pollution data for Germany at a spatial resolution of 2km x 2km were obtained from the German Environment Agency (UBA). The data are based on a data assimilation technique known as Optimal Interpolation (OI). This approach combines air pollution measurements from about 400 background monitoring stations across Germany with the simulated fields of the Chemical transport model REM-CALGRID. Air pollution data, including PM10, PM2.5, NO2, and O3, are available for the years 2004 to 2016.

eTable 1. Synoptic table on spatiotemporal exposure models

| **Country** | **Temperature** | **Type** | **Model description** | **Source of air temperature  observations** | **Source of land surface temperature** | **Main predictors** | **Spatial resolution** | **Temporal resolution** | **Spatial domain** | **Temporal domain** |
| --- | --- | --- | --- | --- | --- | --- | --- | --- | --- | --- |
| Norway | Mean air temperature | Spatial-temporal observational gridded dataset | Spatial interpolation model using observational data | MET Norway's climate database and the European Climate Assessment and Dataset | senorge2 dataset | Temperature and precipitation | 1 km × 1 km | Daily | Norway | 1995-2017 |
| England and Wales | Mean air temperature | Spatial-temporal observational gridded dataset | Spatial interpolation of station temperature accounting for spatial characteristics including land use, altitude and coastal influence. | Met Office HadUK-Grid | Inverse-distance weighted interpolation of station data | Latitude, longitude, altitude, coastal influence and density of urban land use | 1 km × 1 km | Daily | United Kindom (England, Wales, Scotland, Norther Ireland) | 1960-2019 |
| Germany | Mean air temperature (2m above the ground) | Hybrid spatial-temporal  regression-based model | Three-stage approach:  stage 1: linear mixed effects model to calibrate the relationship between observed air temperature and satellite-derived land surface temperature stage 2: predict Ta in grid cells without air temperature observations but with land surface temperature available stage 3: mixed effects model of stage-2 predictions against interpolated air temperature observations based a thin plate spline | 406 monitoring stations of the National Meteorological Service of Germany (DWD) | MOD11A1 product of MODIS spectroradiometer of Terra satellite | Satellite-based land surface temperature, elevation, NDVI, percentages of urban fabric, arable land, pastures, forests, and inland waters | 1 km × 1 km | Daily | Germany | 2000-2019 |

eTable 2. Definition of territorial typologies by EUROSTAT

| Urban type | Predominantly urban regions: the share of the population living in rural areas is below 20% |
| --- | --- |
|  | **Intermediate regions**: the share of the population living in rural areas is between 20%and 50% |
|  | **Predominantly rural regions**: the share of the population living in rural areas is higher than 50%, where 'rural areas' are all areas outside urban clusters and 'urban clusters' are clusters of contiguous grid cells of 1 km² with a density of at least 300 inhabitants per km² and a minimum population of 5,000) |
| Mountain type | > 50% of population live in mountain areas; 2: > 50% of surface in mountain area |
|  | > 50% of surface in mountain area |
|  | > 50% of population live and 50% of surface in mountain areas |
|  | Non-mountain region |
| Coastal type | Sea border |
|  | > 50% of the population within 50 km of coastline |
|  | Non-coastal |

eFigure 1. Exposure-response functions for temperature-respiratory mortality associations in four European countries (black dashed line: Minimum Mortality Temperature; red dashed line: 99th percentile of the temperature distribution)

| **Norway** | **England and Wales** |
| --- | --- |
| 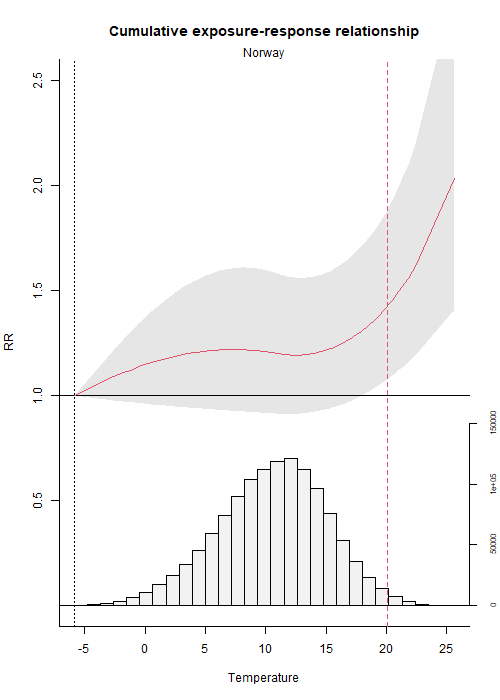 | 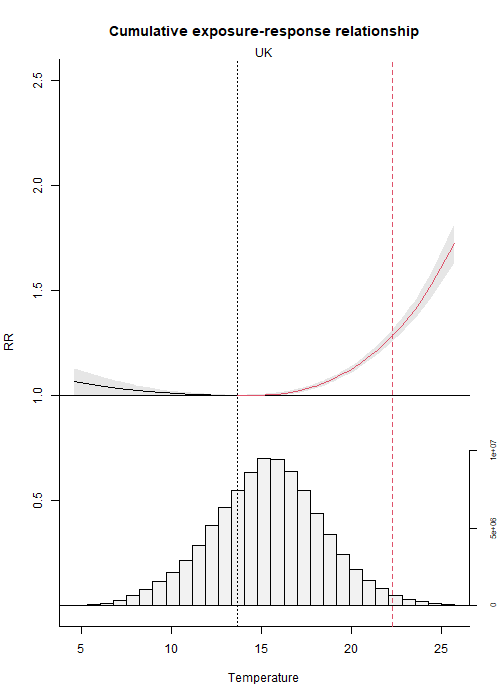 |
| **Germany** | 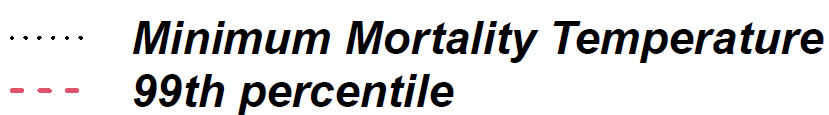 |
| 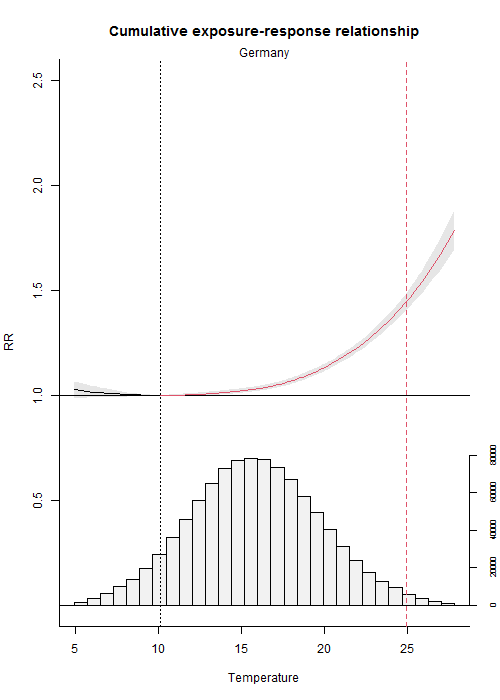 |  |

eTable 3. Relative Risks (and 95% Confidence Intervals) per increase in the two-day mean temperature from the 75th to the 99th percentile of the area-specific distribution (Main analysis) and corresponding results from the sensitivity analyses and by subgroups by country

|  | Norway | England and Wales | Germany | Overall |
| --- | --- | --- | --- | --- |
| Main model | 1.19 (1.11, 1.27) | 1.25 (1.23, 1.28) | 1.34 (1.3, 1.38) | 1.27 (1.19, 1.34) |
| Different def. of warm period | 1.25 (1.15, 1.35) | 1.25 (1.23, 1.28) | 1.35 (1.31, 1.4) | 1.29 (1.21, 1.36) |
| Lag 0-3 | 1.23 (1.14, 1.32) | 1.29 (1.25, 1.32) | 1.45 (1.4, 1.51) | 1.32 (1.21, 1.45) |
| 99th pct versus MMT | 1.42 (1.07, 1.88) | 1.28 (1.26, 1.31) | 1.44 (1.4, 1.49) | 1.36 (1.23, 1.52) |
| 65+ years | 1.2 (1.12, 1.28) | 1.26 (1.23, 1.29) | 1.35 (1.31, 1.39) | 1.28 (1.20, 1.35) |
| Males | 1.17 (1.07, 1.28) | - | 1.3 (1.26, 1.35) | 1.24 (1.12, 1.38) |
| Females | 1.2 (1.09, 1.32) | - | 1.38 (1.32, 1.44) | 1.30 (1.13, 1.49) |

eTable 4. Descriptive statistics of the vulnerability factors and other potential effect modifiers at NUTS 3 level by country

| **Vulnerability factor/ Potential effect modifier** | **Country (number of NUTS 3 areas)** | **5^th^ perc** | **25^th^ perc** | **Median** | **75^th^ perc** | **95^th^ perc** |
| --- | --- | --- | --- | --- | --- | --- |
| **Population density (inhabitants per km^2^)** | **Germany (380)** | 74.00 | 120.00 | 203.00 | 661.00 | 2059.00 |
|  | **Norway (11)** | 3.00 | 7.00 | 17.00 | 45.00 | 1328.00 |
|  | **England and Wales (145)** | 94.00 | 306.00 | 673.00 | 3128.00 | 8869.00 |
| **Urbanized area (%)** | **Germany (380)** | 3.00 | 5.00 | 7.00 | 16.00 | 35.00 |
|  | **Norway (11)** | 0.00 | 0.00 | 1.00 | 2.00 | 24.00 |
|  | **England and Wales (145)** | 2.00 | 7.00 | 16.00 | 55.00 | 79.00 |
| **Green area per 100.000 inhabitants** | **Germany (380)** | 21.97 | 112.22 | 452.03 | 767.69 | 1265.64 |
|  | **Norway (11)** | 50.16 | 1782.70 | 3373.51 | 6844.14 | 13408.38 |
|  | **England and Wales (145)** | 0.78 | 9.69 | 109.77 | 276.04 | 1025.69 |
| **Population aged over 65 years (%)** | **Germany (380)** | 17.71 | 19.35 | 20.60 | 21.94 | 24.56 |
|  | **Norway (11)** | 12.34 | 15.68 | 15.83 | 17.66 | 19.59 |
|  | **England and Wales (145)** | 11.34 | 14.80 | 18.34 | 20.74 | 25.00 |
| **Gross domestic product at current market prices (€)** | **Germany (380)** | 15550.00 | 19835.29 | 25120.00 | 29140.00 | 35380.00 |
|  | **Norway (11)** | 40088.89 | 45866.67 | 47244.45 | 56211.11 | 94088.89 |
|  | **England and Wales (145)** | 18321.67 | 21258.97 | 25691.34 | 30960.00 | 48813.24 |
| **Ratio of employed population and population aged 20-64 (%)** | **Germany (380)** | 56.72 | 70.00 | 77.28 | 89.66 | 130.28 |
|  | **Norway (11)** | 36.60 | 38.85 | 42.77 | 44.37 | 84.36 |
|  | **England and Wales (145)** | 66.35 | 73.10 | 76.92 | 80.56 | 83.65 |
| **PM_2.5_(μg/m^3^)** | **Germany (380)** | 10.01 | 11.36 | 11.95 | 13.13 | 15.46 |
|  | **Norway (11)** | 2.26 | 2.99 | 4.18 | 4.76 | 6.75 |
|  | **England and Wales (145)** | 9.23 | 11.27 | 12.54 | 13.61 | 15.73 |

| **Urban type** | **Predominantly urban regions** | **Intermediate regions** | **Predominantly rural regions** |  |
| --- | --- | --- | --- | --- |
| **Germany** | 24.13 % | 47.99 % | 27.88 % |  |
| **Norway** | 11.11 % | 44.44 % | 44.44 % |  |
| **England and Wales** | 75.86 % | 19.31 % | 4.83 % |  |
| **Mountain type** | > 50% of population live in mountain areas | > 50% of surface in mountain area | > 50% of population live and 50% of surface in mountain areas | Non-mountain region |
| **Germany** | 0.53 % | 4.22 % | 7.65 % | 87.60 % |
| **Norway** | 0.00 % | 50.00 % | 37.50 % | 12.50 % |
| **England and Wales** | 0.00 % | 3.45 % | 0.69 % | 95.86 % |
| **Coastal type** | Sea border | > 50% of the population within 50 km of coastline | Non-coastal |  |
| **Germany** | 5.29 % | 2.38 % | 92.33 % |  |
| **Norway** | 90.91 % | 0.00 % | 9.09 % |  |
| **England and Wales** | 50.34 % | 29.66 % | 20.00 % |  |

eTable 5: Percent of NUTS3 areas by country and region type

eTable 6: Spearman correlation coefficients of the vulnerability factors and other potential effect modifiers in Germany

| **Germany** | **Population density** | **Urbanized area (%)** | **Green area per 100.000 inhabitants** | **% of population aged over 65 years** | **Gross domestic product** | **Ratio of employed population and population aged 20-64 (%)** | **PM_2.5_** |
| --- | --- | --- | --- | --- | --- | --- | --- |
| **Population density (inhabitants per km^2^)** | 1.00 |  |  |  |  |  |  |
| **Urbanized area (%)** | 0.96 | 1.00 |  |  |  |  |  |
| **Green area per 100.000 inhabitants** | -0.99 | -0.97 | 1.00 |  |  |  |  |
| **% of population aged over 65 years** | -0.12 | -0.03 | 0.10 | 1.00 |  |  |  |
| **Gross domestic product at current market prices (€)** | 0.61 | 0.55 | -0.61 | -0.27 | 1.00 |  |  |
| **Ratio of employed population and population aged 20-64 (%)** | 0.47 | 0.44 | -0.47 | 0.08 | 0.82 | 1.00 |  |
| **PM_2.5_** (μg/m^3^) | 0.56 | 0.60 | -0.56 | -0.08 | 0.23 | 0.16 | 1.00 |

eTable 7: Spearman correlation coefficients of the vulnerability factors and other potential effect modifiers in Germany

| **Germany** | **Population density** | **Urbanized area (%)** | **Green area per 100.000 inhabitants** | **% of population aged over 65 years** | **Gross domestic product** | **Ratio of employed population and population aged 20-64 (%)** | **PM_2.5_** |
| --- | --- | --- | --- | --- | --- | --- | --- |
| **Population density (inhabitants per km^2^)** | 1.00 |  |  |  |  |  |  |
| **Urbanized area (%)** | 0.96 | 1.00 |  |  |  |  |  |
| **Green area per 100.000 inhabitants** | -0.99 | -0.97 | 1.00 |  |  |  |  |
| **% of population aged over 65 years** | -0.12 | -0.03 | 0.10 | 1.00 |  |  |  |
| **Gross domestic product at current market prices (€)** | 0.61 | 0.55 | -0.61 | -0.27 | 1.00 |  |  |
| **Ratio of employed population and population aged 20-64 (%)** | 0.47 | 0.44 | -0.47 | 0.08 | 0.82 | 1.00 |  |
| **PM_2.5_** (μg/m^3^) | 0.56 | 0.60 | -0.56 | -0.08 | 0.23 | 0.16 | 1.00 |

*eTable* 8: Spearman correlation coefficients of the vulnerability factors and other potential effect modifiers in Norway

| **Norway** | **Population density** | **Urbanized area (%)** | **Green area per 100.000 inhabitants** | **% of population aged over 65 years** | **Gross domestic product** | **Ratio of employed population and population aged 20-64 (%)** | **PM_10_** | **PM_2.5_** | **NO_2_** |
| --- | --- | --- | --- | --- | --- | --- | --- | --- | --- |
| **Population density (inhabitants per km2)** | 1.00 |  |  |  |  |  |  |  |  |
| **Urbanized area (%)** | 0.98 | 1.00 |  |  |  |  |  |  |  |
| **Green area per 100.000 inhabitants** | -0.95 | -0.91 | 1.00 |  |  |  |  |  |  |
| **% of population aged over 65 years** | -0.62 | -0.57 | 0.65 | 1.00 |  |  |  |  |  |
| **Gross domestic product at current market prices (€)** | 0.53 | 0.45 | -0.68 | -0.85 | 1.00 |  |  |  |  |
| **Ratio of employed population and population aged 20-64 (%)** | -0.10 | -0.20 | -0.09 | -0.20 | 0.45 | 1.00 |  |  |  |
| **PM_10_ (μg/m^3^)** | 0.65 | 0.58 | -0.81 | -0.65 | 0.79 | 0.55 | 1.00 |  |  |
| **PM_2.5_ (μg/m^3^)** | 0.94 | 0.95 | -0.85 | -0.64 | 0.50 | -0.09 | 0.64 | 1.00 |  |
| **NO_2_ (μg/m^3^)** | 0.94 | 0.92 | -0.94 | -0.48 | 0.46 | 0.09 | 0.74 | 0.87 | 1.00 |

eTable 9: Spearman correlation coefficients of the vulnerability factors and other potential effect modifiers in England and Wales

| **England and Wales** | **Population density** | **Urbanized area (%)** | **Green area per 100.000 inhabitants** | **% of population aged over 65 years** | **Gross domestic product** | **Ratio of employed population and population aged 20-64 (%)** | **PM_2.5_** |
| --- | --- | --- | --- | --- | --- | --- | --- |
| **Population density (inhabitants per km2)** | 1.00 |  |  |  |  |  |  |
| **Urbanized area (%)** | 0.99 | 1.00 |  |  |  |  |  |
| **Green area per 100.000 inhabitants** | -0.99 | -0.99 | 1.00 |  |  |  |  |
| **% of population aged over 65 years** | -0.80 | -0.77 | 0.79 | 1.00 |  |  |  |
| **Gross domestic product at current market prices (€)** | 0.27 | 0.26 | -0.26 | -0.49 | 1.00 |  |  |
| **Ratio of employed population and population aged 20-64 (%)** | -0.50 | -0.50 | 0.52 | 0.37 | 0.25 | 1.00 |  |
| **PM_2.5_ (μg/m^3^)** | 0.67 | 0.65 | -0.65 | -0.7 | 0.47 | 0.00 | 1.00 |

eTable 10: Relative Risks (and 95% CIs) for the effect on respiratory mortality for an increase in temperature from the Minimum Mortality Temperature (9.0 ^o^C) to the 99th percentile (22.4 ^o^C) at the low and high level of the effect modifier (5^th^ and 95^th^ percentile respectively):

|  | **5^th^ percentile** | **RR (95 % CI)** | |
| --- | --- | --- | --- |
|  | **95^th^ percentile** |  |  |
|  |  | **Unadjusted models** | **Adjusted for population density** |
| Population density (inhabitants per km^2^) | 65.00 | 1.26 (1.19, 1.33)^*^ |  |
|  | 3942.00 | 1.41 (1.33, 1.50) ^*^ |  |
| Urbanized area (%) | 3.00 | 1.25 (1.18, 1.32) ^*^ |  |
|  | 66.00 | 1.44 (1.35, 1.54) ^*^ |  |
| Green area per 100,000 inhabitants | 4.37 | 1.33 (1.27, 1.38) |  |
|  | 1447.14 | 1.28 (1.23, 1.34) |  |
| Gross domestic product at current market prices (€) | 17570.59 | 1.29 (1.24, 1.35) | 1.27 (1.20, 1.34) |
|  | 54541.18 | 1.33 (1.27, 1.39) | 1.25 (1.18, 1.33) |
| Ratio of employed population and population aged 20-64 (%) | 56.40 | 1.27 (1.22, 1.33) | 1.24 (1.18, 1.32) |
|  | 122.50 | 1.37 (1.29, 1.46) | 1.31 (1.22, 1.41) |
| Population aged over 65 years (%) | 13.36 | 1.38 (1.28, 1.48) ^*^ | 1.27 (1.18, 1.37) |
|  | 24.68 | 1.21 (1.13, 1.30) ^*^ | 1.26 (1.18, 1.34) |
| PM_25_ (μg/m^3^) | 9.23 | 1.21 (1.16, 1.26) ^*^ | 1.22 (1.16, 1.28)^*^ |
|  | 15.57 | 1.45 (1.38, 1.51) ^*^ | 1.39 (1.30, 1.47) ^*^ |
|  |  |  |  |
|  | **Category** |  |  |
| Urban type | Predominantly urban regions | 1.35 (1.27, 1.43) ^*^ |  |
|  | Intermediate regions | 1.24 (1.16, 1.32) ^*^ |  |
|  | Predominantly rural regions | 1.25 (1.16, 1.35) ^*^ |  |
| Mountain type | > 50% of population live in mountain areas | 1.30 (0.92, 1.83) | 1.26 (0.90, 1.77) |
|  | > 50% of surface in mountain area | 1.24 (1.13, 1.36) | 1.23 (1.12, 1.36) |
|  | > 50% of population live and 50% of surface in mountain areas | 1.21 (1.10, 1.33) | 1.16 (1.04, 1.28) |
|  | Non-mountain region | 1.32 (1.27, 1.37) | 1.27 (1.20, 1.35) |
| Coastal type | Sea border | 1.27 (1.22, 1.33) | 1.23 (1.16, 1.30) |
|  | > 50% of the population within 50 km of coastline | 1.35 (1.28, 1.42) | 1.30 (1.22, 1.39) |
|  | Non-coastal | 1.32 (1.28, 1.37) | 1.28 (1.22, 1.35) |
| * :statistically significant effect modifier at 5% level of the heat-mortality association based on the Wald test | | | |

eTable 11: Relative Risks (and 95% CIs) for the effect on respiratory mortality for an increase in temperature from the 95^th^ to the 99th percentile (22.4 ^o^C) at the low and high level of the effect modifier (5^th^ and 95^th^ percentile respectively) by country:

|  |  |  | **RR (95 % CI)** | |
| --- | --- | --- | --- | --- |
|  |  |  |  |  |
|  |  | **5^th^ percentile** | **Unadjusted models** | **Adjusted for population density** |
|  |  | **95^th^ percentile** |  |  |
| Population density (inhabitants per km^2^) | Germany | 74.00 | 1.28 (1.24- 1.33)^*^ |  |
|  |  | 2059.00 | 1.45 (1.39- 1.52) ^*^ |  |
|  | Norway | 3.00 | 1.17 (1.09- 1.25) |  |
|  |  | 1328.00 | 1.25 (1.13- 1.39) |  |
|  | England and Wales | 94.00 | 1.22 (1.18- 1.26) ^*^ |  |
|  |  | 8869.00 | 1.40 (1.29- 1.51) ^*^ |  |
| Urbanized area (%) | Germany | 3.00 | 1.27 (1.22- 1.32) ^*^ |  |
|  |  | 35.00 | 1.47 (1.4- 1.54) ^*^ |  |
|  | Norway | 0.00 | 1.16 (1.08- 1.25) |  |
|  |  | 24.00 | 1.26 (1.13- 1.40) |  |
|  | England and Wales | 2.00 | 1.23 (1.18- 1.27) ^*^ |  |
|  |  | 79.00 | 1.30 (1.23- 1.38) ^*^ |  |
| Green area per 100,000 inhabitants | Germany | 21.97 | 1.42 (1.37- 1.47) ^*^ |  |
|  |  | 1265.64 | 1.18 (1.11- 1.26) ^*^ |  |
|  | Norway | 50.16 | 1.26 (1.15- 1.39) |  |
|  |  | 13408.38 | 1.02 (0.87- 1.21) |  |
|  | England and Wales | 0.78 | 1.24 (1.20- 1.28) |  |
|  |  | 1025.69 | 1.30 (1.23- 1.37) |  |
| Gross domestic product at current market prices (€) | Germany | 15550.00 | 1.28 (1.24- 1.33) ^*^ | 1.28 (1.23- 1.33) |
|  |  | 35380.00 | 1.45 (1.37- 1.530) ^*^ | 1.34 (1.24- 1.44) |
|  | Norway | 40088.89 | 1.15 (1.06- 1.24) | 1.16 (1.05- 1.28) |
|  |  | 94088.89 | 1.27 (1.12- 1.44) | 1.21 (0.84- 1.74) |
|  | England and Wales | 18321.67 | 1.25 (1.21- 1.28) | 1.23 (1.2- 1.27) |
|  |  | 48813.24 | 1.26 (1.22- 1.30) | 1.22 (1.17- 1.26) |
| Ratio of employed population and population aged 20-64 (%) | Germany | 56.72 | 1.29 (1.23- 1.35) | 1.29 (1.23- 1.36) |
|  |  | 130.28 | 1.43 (1.32- 1.53) | 1.29 (1.18- 1.41) |
|  | Norway | 36.60 | 1.20 (1.12- 1.3) | 1.19 (1.10- 1.28) |
|  |  | 84.36 | 1.11 (0.91- 1.34) | 1.02 (0.83- 1.27) |
|  | England and Wales | 66.35 | 1.21 (1.15- 1.28) | 1.14 (1.07- 1.21) |
|  |  | 83.65 | 1.28 (1.23- 1.34) | 1.28 (1.23- 1.34) |
| Population aged over 65 years (%) | Germany | 17.71 | 1.39 (1.33- 1.46) | 1.32 (1.25- 1.40) |
|  |  | 24.56 | 1.27 (1.20- 1.34) | 1.26 (1.19- 1.34) |
|  | Norway | 12.34 | 1.24 (1.10- 1.41) | 1.17 (0.97- 1.40) |
|  |  | 19.59 | 1.14 (1.02- 1.27) | 1.17 (1.03- 1.32) |
|  | England and Wales | 11.34 | 1.31 (1.25- 1.38) ^*^ | 1.23 (1.14- 1.33) |
|  |  | 25.00 | 1.19 (1.14- 1.26) ^*^ | 1.23 (1.16- 1.3) |
| PM_2.5_ (μg/m^3^) | Germany | 10.01 | 1.20 (1.14- 1.25) ^*^ | 1.19 (1.13- 1.25) ^*^ |
|  |  | 15.46 | 1.49 (1.43- 1.55) ^*^ | 1.51 (1.40- 1.63) ^*^ |
|  | Norway | 2.26 | 1.06 (0.93- 1.21) | 1.05 (0.90- 1.23) |
|  |  | 6.75 | 1.30 (1.16- 1.46) | 1.32 (1.1- 1.58) |
|  | England and Wales | 9.23 | 1.18 (1.12- 1.25) ^*^ | 1.22 (1.15- 1.29) |
|  |  | 15.73 | 1.33 (1.26- 1.40) ^*^ | 1.25 (1.15- 1.36) |

|  |  | **Category** | **RR (95 % CI)** | |
| --- | --- | --- | --- | --- |
|  |  |  | **Unadjusted models** | **Adjusted for population density** |
| Urban type | Germany | Predominantly urban regions | 1.45 (1.39- 1.51) ^*^ |  |
|  |  | Intermediate regions | 1.28 (1.23- 1.33) ^*^ |  |
|  |  | Predominantly rural regions | 1.28 (1.20- 1.36) ^*^ |  |
|  | Norway | Predominantly urban regions | 1.34 (1.10- 1.63) |  |
|  |  | Intermediate regions | 1.16 (1.06- 1.28) |  |
|  |  | Predominantly rural regions | 1.11 (0.96- 1.29) |  |
|  | England and Wales | Predominantly urban regions | 1.25 (1.22- 1.29) |  |
|  |  | Intermediate regions | 1.21 (1.14- 1.29) |  |
|  |  | Predominantly rural regions | 1.48 (1.29- 1.68) |  |
| Mountain type | Germany | > 50% of population live in mountain areas | 1.63 (1.10- 2.40) | 1.61 (1.09- 2.38) |
|  |  | > 50% of surface in mountain area | 1.32 (1.14- 1.52) | 1.31 (1.14- 1.51) |
|  |  | > 50% of population live and 50% of surface in mountain areas | 1.15 (1.02- 1.29) | 1.15 (1.02- 1.29) |
|  |  | Non-mountain region | 1.35 (1.31- 1.39) | 1.30 (1.26- 1.34) |
|  | Norway | > 50% of population live in mountain areas |  |  |
|  |  | > 50% of surface in mountain area | 1.18 (1.05- 1.32) | 1.10 (0.96- 1.27) |
|  |  | > 50% of population live and 50% of surface in mountain areas | 1.21 (1.04- 1.40) | 1.21 (1.04- 1.40) |
|  |  | Non-mountain region | 1.24 (1.08- 1.42) | 1.23 (1.07- 1.41) |
|  | England and Wales | > 50% of population live in mountain areas |  |  |
|  |  | > 50% of surface in mountain area | 1.29 (1.10- 1.51) | 1.29 (1.10- 1.51) |
|  |  | > 50% of population live and 50% of surface in mountain areas | 1.19 (0.88- 1.61) | 1.19 (0.88- 1.61) |
|  |  | Non-mountain region | 1.25 (1.22- 1.29) | 1.25 (1.22- 1.29) |
| Coastal type | Germany | Sea border | 1.22 (1.09- 1.36) | 1.18 (1.06- 1.32) |
|  |  | > 50% of the population within 50 km of coastline | 1.12 (0.91- 1.39) | 1.1 (0.89- 1.36) |
|  |  | Non-coastal | 1.35 (1.31- 1.39) | 1.31 (1.26- 1.35) |
|  | Norway | Sea border | 1.18 (1.11- 1.27) | 1.16 (1.08- 1.25) |
|  |  | > 50% of the population within 50 km of coastline |  |  |
|  |  | Non-coastal | 1.21 (0.99- 1.47) | 1.21 (1.00- 1.47) |
|  | England and Wales | Sea border | 1.26 (1.21- 1.30) | 1.26 (1.21- 1.30) |
|  |  | > 50% of the population within 50 km of coastline | 1.29 (1.23- 1.35) | 1.29 (1.23- 1.35) |
|  |  | Non-coastal | 1.20 (1.13- 1.26) | 1.20 (1.13- 1.26) |
| * :statistically significant effect modifier at 5% level of the heat-mortality association based on the Wald test | | | | |
